# Supplementary figures and images for: RNA-directed DNA methylation prevents rapid and heritable reversal of transposon silencing under heat stress in Zea mays
Source: PLoS Genet. 2021 Jun 14;17(6):e1009326. doi: 10.1371/journal.pgen.1009326 (PMC8224964; doi:10.1371/journal.pgen.1009326)

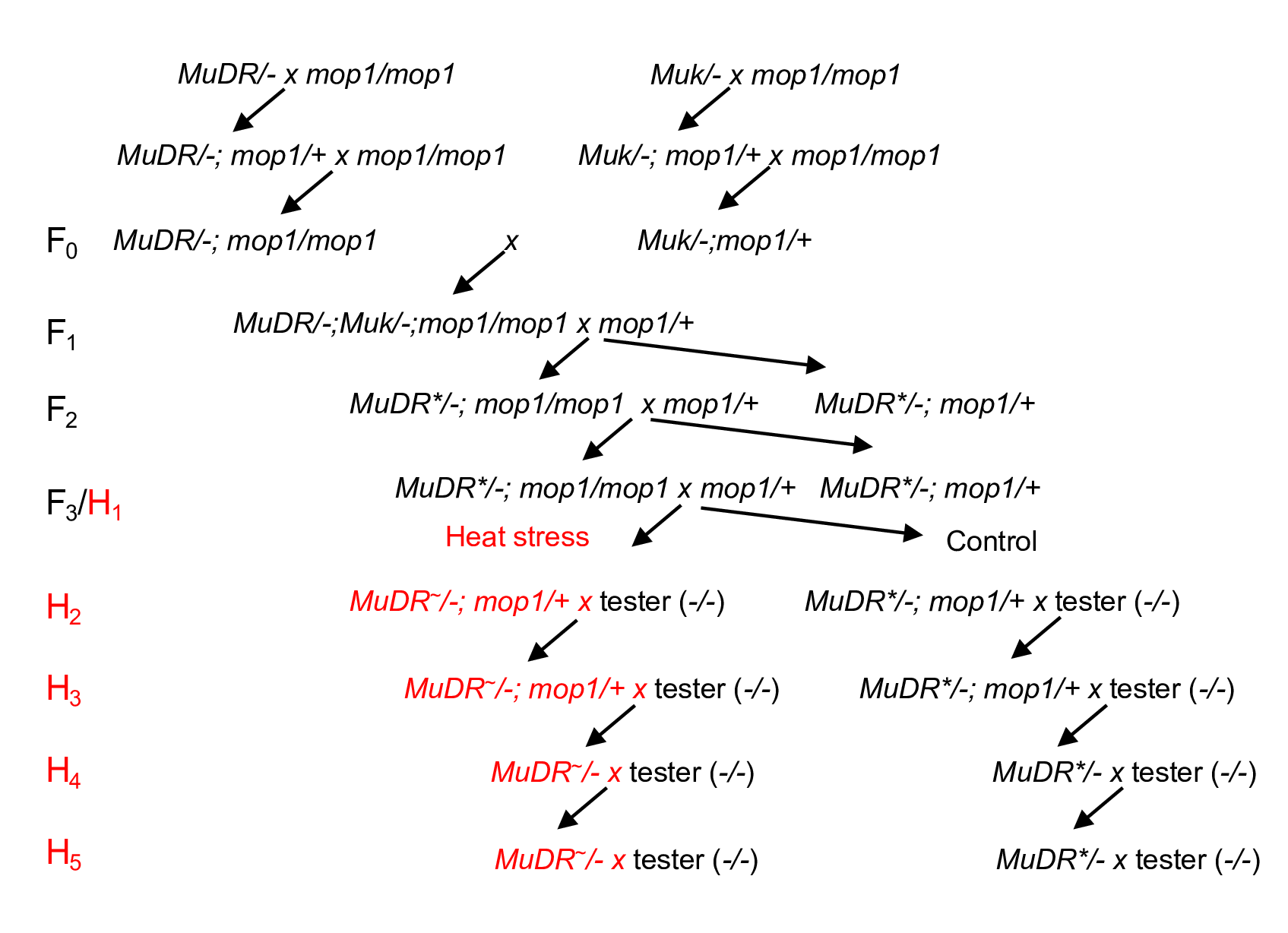

Supplement: S1 Fig — F1 refers to the first generation during which MuDR was exposed to Muk. H1, which corresponds to F3, is the generation in which a brief heat treatment was applied. MuDR indicates an active MuDR element. MuDR* indicates an inactive MuDR element. MuDR~ indicates a reactivated MuDR element. Red text indicates a sample that has been heat-reactivated. (TIF) [file pgen.1009326.s001.tif]

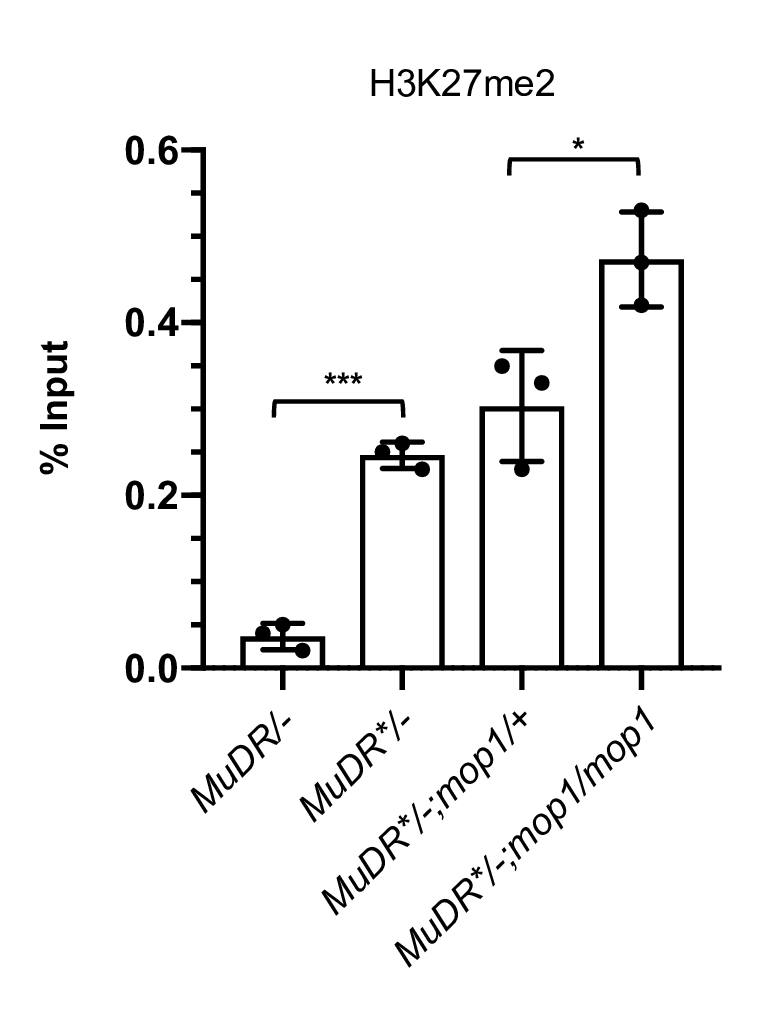

Supplement: S2 Fig — Relative enrichment of H3K27me2 at TIRA in leaf 3 of plants of the4 indicated genotypes. The qPCR values were normalized to Copia and then to the value of input samples. An unpaired t-test was performed. Error bars indicate mean ± standard deviation (SD) of the three biological replicates. *P<0.05; ***P<0.001. (TIF) [file pgen.1009326.s002.tif]

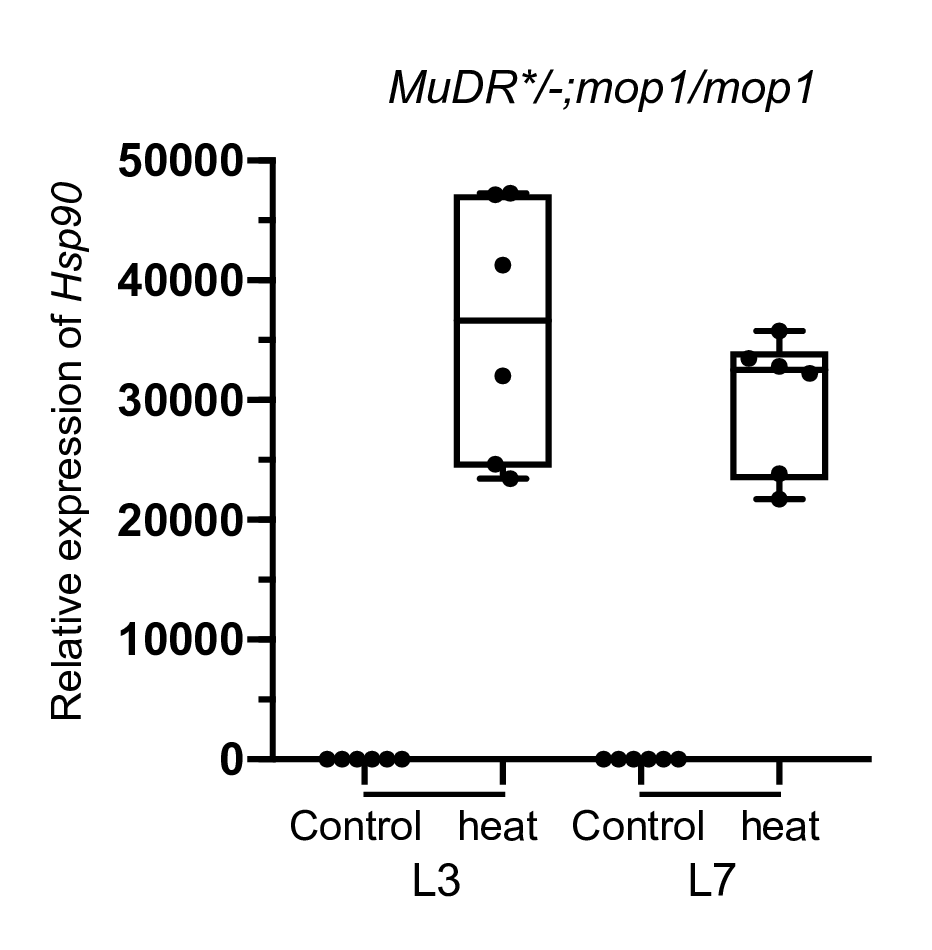

Supplement: S3 Fig — Quantitative real-time PCR was performed to measure transcript levels of Hsp90. Tub2 is used as an internal control gene. For each data point, two of the twelve replicates are pooled. (TIF) [file pgen.1009326.s003.tif]

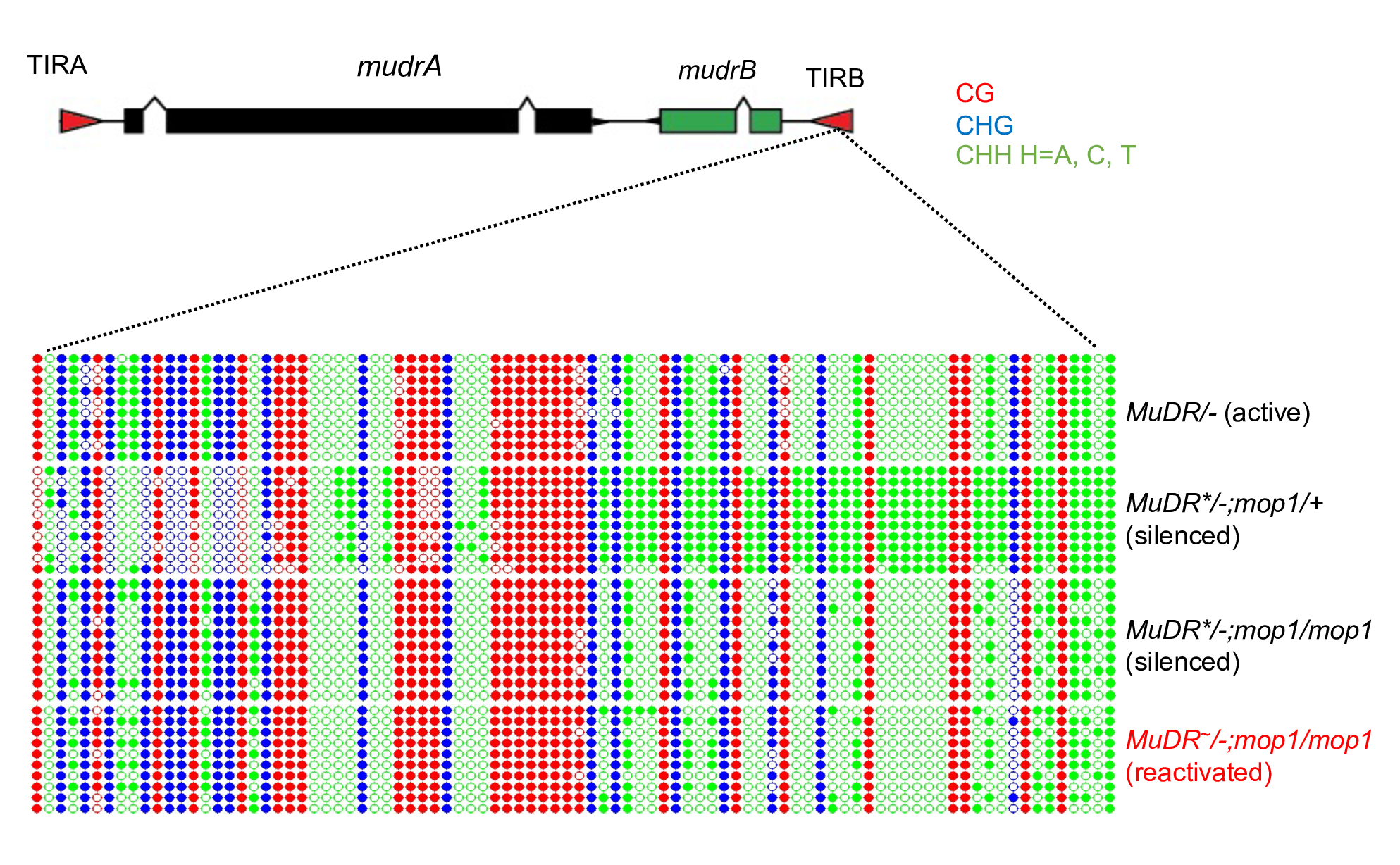

Supplement: S4 Fig — DNA methylation patterns at TIRA and TIRB. Ten individual clones were sequenced from each amplification of bisulfite-treated samples with the indicated genotypes. The cytosines in different sequence contexts are represented by different colors (red, CG; blue, CHG; green, CHH, where H = A, C, or T). For each sample, six independent samples were pooled together. (TIF) [file pgen.1009326.s004.tif]

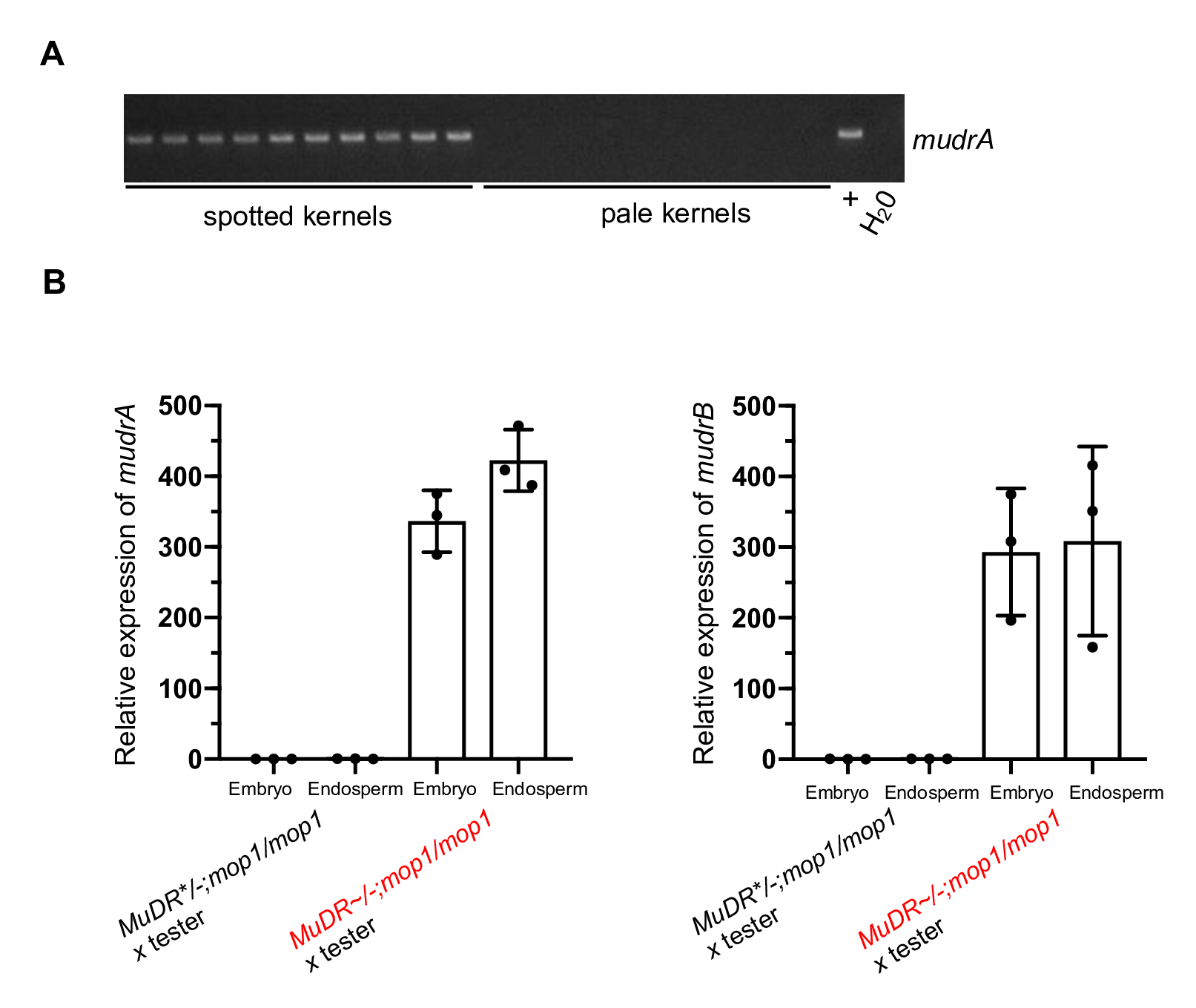

Supplement: S5 Fig — (A) Genotyping results of an ear from the H2 generation. (B) qRT-PCR analysis of mudrA and mudrB expression in embryos and endosperms from kernels derived from three independent ears derived from crosses of H1 heat stressed plants and control. Tub2 is used as an internal control gene. Red text indicates a sample that has been heat-treated. For each family, three independent biological replicates were used. (TIF) [file pgen.1009326.s005.tif]
