# Supplementary material for: RNA-directed DNA methylation prevents rapid and heritable reversal of transposon silencing under heat stress in Zea mays
Source: PLoS Genet. 2021 Jun 14;17(6):e1009326. doi: 10.1371/journal.pgen.1009326 (PMC8224964; doi:10.1371/journal.pgen.1009326)
Supplement: Attachment — Submitted filename: Guo_Response to reviews_R2.docx [file pgen.1009326.s012.docx]

Response to reviews:

The manuscript was fully evaluated at the editorial level and by the previously involved peer reviewers. As you will see, all reviewers agree that the revised version has considered most of the previous concern and support publication. Beside correction of some language error (reviewer 2 and 4), there is only one point that needs another revision: reviewer 2 found some discrepancy between the new data added and the interpretation in the text. We therefore ask you to modify the manuscript accordingly.

Response: We thank editor and reviewers’ input, which has helped us to improve the quality of the manuscript. All the additional experiments suggested by reviewer are indeed interesting. Overall, we suggest that the primary observation, that demethylation follows, rather than proceeds, both histone modification and transcriptional activity in H2 *mop1/+* heterozygous plants, stands on its own. We have modified the text to better reflect the data.

Reviewer #1: The authors have answered to all my comments and I'm satisfied with the revised version of the manuscript. Therefore, I recommend it for publication.

We thank the reviewer for the helpful comments.

Reviewer #2: Guo et al. provides a detailed study of the epigenetic regulation related to the activity of a single Mu element in wild type and mop1 mutant maize plants. The indications of this work that DNA methylation can rather respond to than determine the activity of a locus and that the balance of DNA methylation and histone modifications is dependent on transcriptional activity, adds important information on the variety of mechanisms involved in transgenerational silencing of transposable elements in maize. Especially, it points to new unexplored mechanistic relations and independence respectively between DNA methylation and histone modifications in this context.

In the revised version my comments are adequately addressed, except the concerns raised in the context of the transgenerational adaption of the methylation states to the activity states after stress induced reactivation:

In the analyses of transgenerational stability of the reactivated state for TIRA both MuDR/-; +/+ active and H5 reactivated samples show extreme, fully demethylated DNA. In contrast the heat stressed (H2 reactivated) samples are strongly methylated in all contexts as the silenced MuDR*/-;mop1/+ are. For TIRB similar results were obtained but only related to asymmetric DNA methylation. These results are discussed as an adaption of the methylation state to the activity state of the locus, only after multiple generations. To strengthen this point it would be required to analyze DNA methylation in the generations in between i.e. H3 and H4.

The authors now provide additional data for TIRA and TIRB on H4, which show the same full demethylation as in H5. The text is not changed accordingly (Line 377) to mention the analysis of the H4 generation neither the conclusion that multiple rounds of meiosis are likely required to adapt the methylation profile to the active state. Clearly this conclusion is weakened by the new results, but can be resolved only by analysis of H3 and the heat-stressed, reactivated H1, which the authors do not provide. The new results however indicate that a gradual loss of methylation in adaption to the activity state of the elements over multiple generations is less likely and it is questionable whether meiosis is required or related to the phenomenon at all. Alternatively, the adaption of the methylation state might be independent of meiosis and started already in H1. Indeed, although not quantitatively analyzed, the methylation profiles of reactivated H2 show demethylation compared to the silenced H1 generation. The new results should be described in the text and the interpretations adjusted.

Response: The reviewer makes an excellent point. We agree that we have not shown that switch from methylated to unmethylated is gradual or that it requires multiple rounds of meiosis and have modified our text accordingly.

The new text reads as follows:

Line 380: “we examined TIRA and TIRB methylation in plants three and four generations removed from the initial heat stress.”

Line 383: “This suggests that patterns of methylation consistent with activity are in fact restored in the heat stressed lineage after MOP1 function is restored, but only after changes in H3K9me2 at TIRA and in H3K27me3 at TIRB have already taken place.”

Line 439: “Interestingly, however, at some point after leaf 3 of the H2 generation methylation levels are reduced to those of active *MuDR* elements, suggesting that this reduced methylation level is a consequence, rather than a cause, of maintenance of activity”

Examples of remaining grammatical or typing errors:

Line 386 "latter" instead of "later"

Line 332 "could" is doubled

Line 485 "in maize" instead of "is maize"

Response: These have all been corrected.

Reviewer #3: The authors have taken into consideration all concerns raised by my first report at theoretical level. I think that both introduction and discussion were greatly improved. I understand that the study of their system/model in a different genomic background is not that simple as well as it is not the validation of the results for MuDR at histone modification level. However, I am convinced that these validations are essential to confirm their observations.

Response: We thank the reviewer for the helpful comments.

Reviewer #4: All the major issues are addressed, minor issues are listed below

Line numbers Comment
47 Change out-replicated to out-replicate
145-147 Sentence is confusing, please clarify.
543 Change understanding to understand
566 Remove “for” or “in”
595 Spelling error for collected

Response: We have corrected these errors and clarified the meaning of the indicated passage. It now reads as follows:

Line 145. “Given that various components of regulatory pathways that have evolved to regulate TEs are up-regulated in germinal lineages, it is not surprising that a defect in one of these pathways would lead to an enhancement in the number of germinally transmitted new insertions.”
